# Choledochal Cyst: Is Complete Excision of the Intrapancreatic Cyst Necessar

**DOI:** 10.1155/1998/80404

**Published:** 1998-08

**Authors:** Alastair Millar, Sid Cywes

**Affiliations:** Department Paediatric Surgery Institute of Child Health Red Cross Children's Hospital Rondebosch 7700 South Africa


*HPB Surgery*, 1998, Vol. 11, pp. 61–70
Reprints available directly from the publisher
Photocopying permitted by license only

© 1998 OPA (Overseas Publishers Association) N.V.
Published by license under
the Harwood Academic Publishers imprint,
part of The Gordon and Breach Publishing Group.
Printed in India.

### HPB INTERNATIONAL

**EDITORIAL & ABSTRACTING SERVICE**

JOHN TERBLANCHE EDITOR
Department of Surgery,
Medical School · Observatory 7925
Cape Town · South Africa
Telephone: 27-21-406-6204
Telefax 27-21-448-6461
E-mail: JTER BLAN@UCT GSHI.UCT.AC.2A

### Choledochal Cyst: Is Complete Excision of the Intrapancreatic Cyst Necessary?

### ABSTRACT

*Ando, H., Kaneko, K., Ito, T., Watanabe, Y., Seo, T., Harada, T., Ito, F., Nagaya, M. and Sugito, T. (1996) Complete excision of the intrapancreatic portion of choledochal cysts. Journal of the American College of Surgeons,* ***183****, 317–321.*

***Background:*** **Cyst excision is the treatment for patients with choledochal cysts. In general, many authors recommend intramural dissection between the outer and inner layers of the cyst or partial excision leaving part of the cyst in the pancreas to avoid pancreatic injury. However, because there are few large series with long-term follow-up periods, it remains unclear how much of the intrapancreatic portion of the cyst should be resected and what resection technique should be used.**
***Study Design:*** **During an 18-year period, 104 patients underwent excision of choledochal cysts at our hospitals. Twelve patients had partial excision of the cyst above the pancreas, and 17 had intramural dissection of the intrapancreatic portion. Seventy-five patients underwent complete excision of the intrapancreatic portion of the cyst by our new technique, in which the outer plane of the epicholedochal plexus is dissected, exposing the narrow distal segment connecting the cyst to the pancreatic duct. Our new technique was compared retrospectively with the other two techniques.**
***Results:*** **With our technique, the intrapancreatic cyst could be excised completely in 75 patients without any complications. Blood loss was significantly decreased when our technique was used compared to intramural excision. A pancreatic fistula occurred after intramural excision in one patient, and pancreatic stones formed several years after partial excision and intramural excision in three patients who proved to have residual cystic material in the pancreas.**
***Conclusions:*** **Our operative technique is safe and effective for the complete excision of the intrapancreatic portion of a choledochal cyst. *J. Am. Coll. Surg.*, 1996, 183, 317–321.**

*Keywords:* Choledochal cyst, excision choledochal cyst

### PAPER DISCUSSION

The paper by H. Ando *et al.* reviewed their experience of the management of 104 patients operated on for choledochal cyst over an 18 year period. Seventy-five of these had complete excision of the intra-pancreatic portion of the cyst by a technique in which the outer plane of the epicholedocal plexus is dissected. This method is compared with the technique of intra-mural dissection or partial cyst excision.

Claimed advantages of this technique were, absence of complications, particularly with regard to pancreatic duct injury and decreased blood loss when compared with intra-mural excision. During the follow-up period of 18 years 3 patients developed pancreatic stones in cystic remnants. All three had either an intra-mural dissection or oversewing of a distal cuff of cyst. Presumably either pancreatic secretions or mucin from periductal glands contributed to stone formation as these consisted mainly of protein.

The micro-vascular anatomy of the bile ducts is described with a schematic illustration showing how it is expanded with the development of a choledochal cyst. Thus the intra-pancreatic cyst is shown as having 2 intramural vascular plexuses, the first lying between the outer fibrous connective tissue and the lamina propria and the second in a submucosal plexus. The plane of intra-mural dissection usually adopted to avoid pancreatic injury is that between the lamina propria, much of the mucosa having been replaced with granulation tissue, and the fibrous connective tissue. The point made is that, in their experience, more bleeding resulted from numerous small vessels using the intra-mural technique without the ability of control, whereas dissection external to the cyst allowed for better control of hemorrhage with meticulous ligation of individual vessels and safe complete intra-pancreatic excision.

Management of cystic dilatation of the bile ducts has intrigued general and paediatric surgeons for decades. As this condition is 10 to 20 times more frequently encountered in the Orient, it is not surprising that several of the advances in treatment have come from that quarter. Much has been written of the surgical management of this condition largely because of the high incidence of complications, predominantly bile sludge, gallstones and infection, and in the longterm carcinoma when the cyst is left *in situ*, no matter how efficiently it is drained [1]. Current accepted treatment is complete excision. More emphasis in recent literature has been laid on detail and level of the proximal anastomosis and type of enteric drainage (Roux-en-Y versus doudenal), the width of the anastomosis, and the advisability of hepatic resection in type 1VA cysts, rather than on what to do at the distal end [2]. Ando *et al.* emphasise that radical excision including the distal intra-pancreatic component is both desirable and safe in most cases. The fear of damage to the pancreatic duct during dissection of the distal cyst has encouraged surgeons to either leave some residual intra-pancreatic cyst behind or to perform an intra-mural cystectomy. Ando *et al.* with beautifully clear schematic diagrams describe their technique whereby the distal cyst is completely excised in a plane outside the cyst wall. The multiple vessels arising from the pancreatico-duodenal arteries are easily identified and individually ligated and divided. Another important observation they have made is that the narrowed distal bile duct is located not at the most caudal extremity of the cyst but more proximally on the right ventral aspect of the cyst. Miyano has advocated intra-operative cyst endoscopy to examine the proximal bile duct but this technique could just as well be used to identify the exit site of the distal duct thus helping identification at surgery [3]. Reported early complications of the distal dissection are indeed that of either pancreatitis or pancreatic fistula from damage to the pancreatic ducts, but must be relatively uncommon with minor morbidity as there has been little comment outside the Japanese literature. Indeed, from personal experience of complete distal excision in the plane described, there have been no early complications, which has surprised us, as often scattered pancreatic tissue is splayed out over the the inferior aspect of the cyst and is left *in-situ* at the completion of the operation. Late complications in the form of pancreatic stones in three of their patients with the finding of residual cyst in all, emphasises that complete exterpation of the cyst is an essential component to the operation. Although malignancy was not found in their series, this has been described arising in the terminal pancreatic portion of the cyst 12 years after excision [4]. Complete cyst excision can be safely performed in nearly all cases and is now well established as the treatment of choice for choledochal cysts. A good case can be made to extend total excision to include the intrapancreatic portion of the cyst without fear of pancreatic duct damage. The entire cyst is removed thereby eliminating the chance of later malignancy.

### *References*

[1] Todani, Y. *et al.* (1995). Biliary Complications after Excisional Procedure for Choledochal Cyst. *J. Pediatr. Surg.*, **30**, 478 – 481.

[2] Ohi, R. *et al.* (1990). Surgical Treatment of Congenital Dilatation of the Bile Duct with Special Reference to Late Complications after Total Excisional Operation. *J. Pediatr. Surg.*, **25**, 613 – 617.

[3] Miyano, T. *et al.* (1996). Hepaticoenterostomy after Excision of Choledochal Cyst in Children: A 30 year Experience with 180 cases. *J. Pediatr Surg.*, **31**, 1417–1421.

[4] Yoshikawa, K., Yoshida, K. and Shirai, Y. *et al.* (1986). A case of carcinoma arising in the intrapancreatic terminal choledochus 12 years after primary excision of a giant choledochal cyst. *Am. J. Gastroenterol*, **81**, 378 – 384.

Alastair Millar
Sid Cywes
Department Paediatric Surgery
Institute of Child Health
Red Cross Children's Hospital
Rondebosch 7700
South Africa

### Can Hepatocellular Carcinoma be Cured by Microwave Coagulation?

### ABSTRACT

*Sato, M., Watanabe, Y., Ueda, S., Iseki, S., Abe, Y., Sato, N., Kimura, S., Okubo, K. and Onji, M. (1996) Microwave coagulation therapy for hepatocellular carcinoma. Gastroenterology; 110, 1507 – 1514.*

***Background and Aims:*** **Surgical resection is not always feasible in patients with hepatocellular carcinoma. Microwave coagulation therapy has been used as an alternative to resection, and its efficacy has been evaluated.**
***Methods:*** **Nineteen patients with unresectable hepatocellular carcinoma underwent microwave coagulation therapy through laparotomy (n=12), laparoscopy (n=5), or thoracotomy (n=2) because of advanced liver cirrhosis and/or intrahepatic metastases. One nodule was treated in 13 patients, and two to five nodules were treated in 6 patients; tumor size ranged from 5 to 90 mm. Patient outcomes were studied.**
***Results:*** **Microwave coagulation therapy created a reproducible regional necrosis. Fourteen patients underwent potentially curative treatment; the remaining 5 patients underwent palliative treatment (n = 4) or incomplete tumor coagulation (n = 1). Of the 31 nodules treated, 28 underwent complete tumor ablation. Only 2 patients undergoing laparoscopic microwave coagulation therapy developed local recurrence. The coagulated area subsequently shrank. Patients showed rapid recovery without hepatic dysfunction. Thirteen patients, including 2 long-term survivors, are alive either without tumor (n=10; 14 –64 months) or with tumor (n = 3; 17 – 22 months). Six patients died of hepatocellular carcinoma (n=4) or liver insufficiency (n=2).**
***Conclusions*:** **This preliminary study suggests the efficacy of microwave coagulation therapy, including safety and potential curability, in patients with hepatocellular carcinoma with advanced liver cirrhosis and multifocal or central tumors.**

*Keywords:* Hepatocellular carcinoma, microwave coagulation, acetic acid injection

### PAPER DISCUSSION

Hyperthermia has been employed clinically as one of a variety of multi-modal therapies for malignancies. There is increasing evidence to support the hypothesis that cancer cells are more sensitive to heat than normal cells possibly due to alterations in blood supply in neoplastic tissue and a decreased ability for neovascular beds to

